# Whitish daytime radiative cooling using diffuse reflection of non-resonant silica nanoshells

**DOI:** 10.1038/s41598-020-63591-7

**Published:** 2020-04-16

**Authors:** Takahiro Suichi, Atsushi Ishikawa, Takuo Tanaka, Yasuhiko Hayashi, Kenji Tsuruta

**Affiliations:** 1 Department of Electrical and Electronic Engineering, Okayama University, Okayama, Okayama, 700-8530 Japan; 2 Metamaterials Laboratory, RIKEN Cluster for Pioneering Research, Wako Saitama, 351-0198 Japan; 3 Innovative Photon Manipulation Research Team, RIKEN Center for Advanced Photonics, Wako Saitama, 351-0198 Japan; 4 Institute of Post-LED Photonics, Tokushima University, Tokushima, Tokushima, 770-8506 Japan; 5 Present Address: Hamamatsu Photonics K.K., Hamamatsu, Shizuoka Japan

**Keywords:** Metamaterials, Nanophotonics and plasmonics, Metamaterials, Nanoparticles

## Abstract

Daytime radiative cooling offers efficient passive cooling of objects by tailoring their spectral responses, holding great promise for green photonics applications. A specular reflector has been utilized in cooling devices to minimize sunlight absorption, but such a glaring surface is visually less appealing, thus undesirable for public use. Here, by exploiting strong diffuse reflection of silica nanoshells in a polymer matrix, daytime radiative cooling below the ambient temperature is experimentally demonstrated, while showing whitish color under sunlight. The cooling device consists of a poly(methyl methacrylate) layer with randomly distributed silica nanoshells and a polydimethylsiloxane (PDMS) layer on an Ag mirror. The non-resonant nanoshells exhibit uniform diffuse reflection over the solar spectrum, while fully transparent for a selective thermal radiation from the underneath PDMS layer. In the temperature measurement under the sunlight irradiation, the device shows 2.3 °C cooler than the ambient, which is comparable to or even better than the conventional device without the nanoshells. Our approach provides a simple yet powerful nanophotonic structure for realizing a scalable and practical daytime radiative cooling device without a glaring reflective surface.

## Introduction

Tailoring thermal radiation of engineered surfaces offers a promising approach toward green photonics with potential applications in passive cooling, solar energy harvesting, and thermal management^1–6^. In particular, daytime radiative cooling allows a sub-ambient temperature without any external power supply, thereby effectively improving the energy efficiency of building-scale systems, such as air conditioner and photovoltaic cells^7–12^. In such an effective passive cooling, the incident sunlight is completely reflected, while an object is spontaneously cooled by radiative heat exchange with the cold universe through the infrared (IR) atmospheric window. Recent developments of cooling devices have demonstrated 5 ~ 8-°C sub-ambient temperature under the sunlight irradiation by using nanophotonic structure that reflects more than 97% of sunlight and selectively emit mid-infrared (MIR) radiation^13,14^. During the daytime, the incident solar power usually reaches ~1000 Wm^−2^, while the net thermal radiation power can be up to ~100 Wm^−2^ at room temperature; therefore, in order to realize high efficiency cooling down to a sub-ambient temperature, it is essential not only to maximize the MIR radiation but also to minimize the sunlight absorption.

A specular reflector has been utilized in cooling devices to minimize the sunlight absorption, but such a glaring surface is less visually appealing, thus undesirable for public use, such as a building outer wall and automotive body. Although a nanophotonic structure without the strong reflection has been proposed to fairly preserve the color of an object under sunlight, such an approach still suffers from degradation of the cooling performance by the absorption of visible light^15^. On the other hand, a new design approach has recently been proposed for a scalable and efficient radiative cooling by using a randomly distributed optical resonators in a polymer matrix^16^. Such an approach allows for tailoring the spectral response by tuning the refractive index and size parameters of microscopic structure dispersed in the polymer matrix.

Here, we propose a daytime radiative cooling device with randomly distributed silica nanoshells in a PMMA matrix exhibiting a strong diffuse reflection in the visible region. Specifically, we experimentally demonstrate the uniform diffuse reflection of the non-resonant nanoshells over the solar spectrum and a selective thermal radiation from the underneath PDMS layer. The comparable or even better cooling performance of the device is then demonstrated compared to the conventional device with the specular reflection, where the device shows 2.3 °C cooler than the ambient under the sunlight. Our approach provides a scalable structure without glaring reflective surface for realizing a practical daytime radiative cooling.

**Figure 1:**
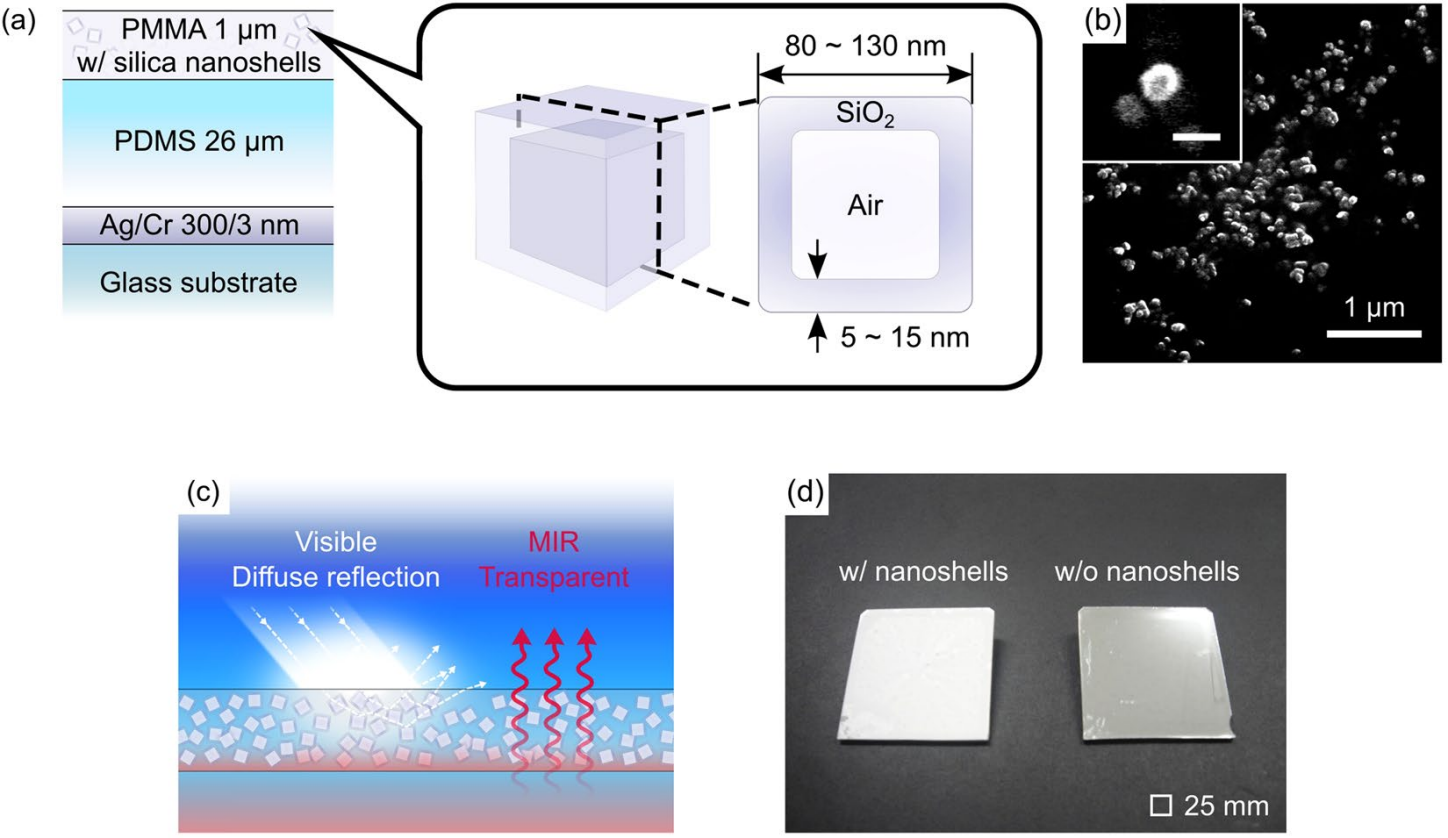
(**a**) Schematic cross-section of a radiative cooling device consisting of a PMMA layer with randomly distributed silica nanoshells and a PDMS layer on a Ag mirror to selectively emit the MIR radiation. (**b**) SEM image of the silica nanoshells and its magnified image in the inset (scale bar: 100 nm). (**c**) The nanoshells diffusely reflect the incident sunlight, but let the MIR radiation transmit through from the underneath PDMS layer. (**d**) Photograph of the fabricated device with (without) the nanoshells on a SiO_2_ substrate. The device exhibits a whitish (silver) color due to strong diffuse (specular) reflection in the visible region.

Figure 1(a) shows a schematic cross-section of a radiative cooling device consisting of a poly(methyl methacrylate) (PMMA) layer with randomly distributed silica nanoshells and a polydimethylsiloxane (PDMS) layer on a Ag mirror. The diameter and shell thickness of the silica nanoshells (Nittetsu Mining Co., Ltd., SiliNax) vary 80 ~ 130 nm and 5 ~ 15 nm, forming air cavities with the pore volume of 9 ~ 13 mL/g. The nanoshells exhibit strong optical scattering in the visible region due to a large refractive index difference between the SiO_2_ shell and the air cavity. The PDMS layer, on the other hand, exhibits a selective MIR radiation in the atmospheric window at λ = 8 ~ 13 μm^17^. Figure 1(b) shows a SEM image of the silica nanoshells and its magnified image, revealing that the diameter of the individual nanoshells is about 100 nm. As shown in Fig. 1(c), the non-resonant nanoshells exhibit uniform diffuse reflection over the solar spectrum, while fully transparent for a selective MIR radiation from the underneath PDMS layer. The Ag mirror reflects a MIR radiation from the PDMS layer to enhance the total thermal radiation upward. Figure 1(d) shows a photograph of the fabricated device with and without the nanoshells on a 25 × 25 mm^2^ SiO_2_ substrate. The fabrication process started with a resistive heating evaporation of a 300-nm Ag film onto the SiO_2_ substrate with a 3-nm Cr adhesion layer. PDMS (Daw Corning, Sylgard 184) was uniformly spin-coated onto a Ag mirror and fully cured at a 75 °C for 2 h. The sample was then completed by spin-coating of a PMMA layer with a silica nanoshells. A device without the nanoshells [the right in Fig. 1(d)] was also prepared for the reference in the same manner. The device with (without) the nanoshells exhibits a whitish (silver) color due to strong diffuse (specular) reflection in the visible region.

**Figure 2:**
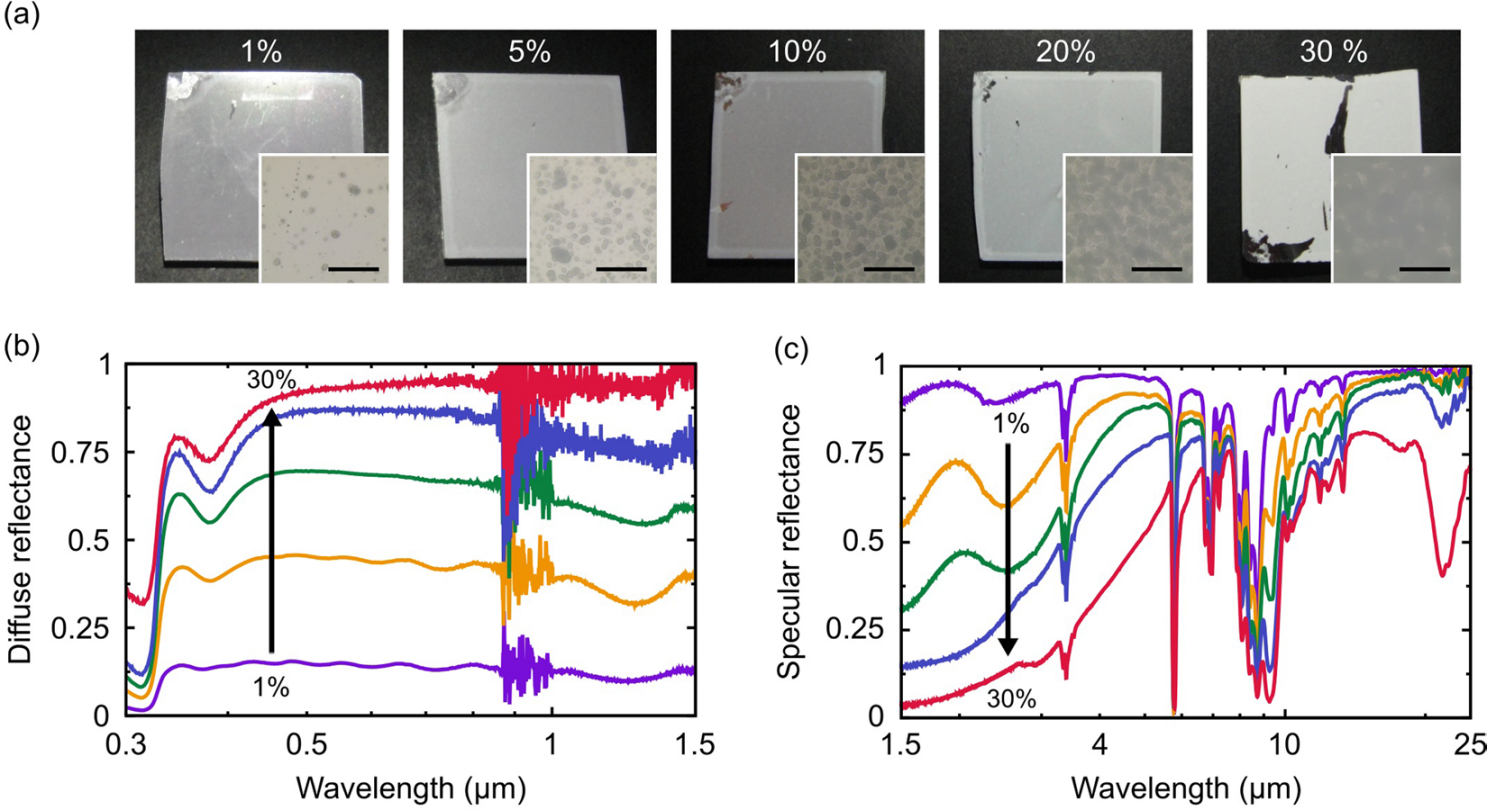
(**a**) Photographs of PMMA films on an Ag mirror by progressively increasing the concentration of the nanoshells in PMMA solution. The insets are reflection microscope images of the films, revealing large aggregations of the nanoshells (scale bar: 20 μm). Experimentally measured (**b**) UV-visible-NIR diffuse reflectance and (**c**) MIR specular reflectance of the PMMA films on the Ag mirror.

To quantitatively investigate the diffuse reflection of the non-resonant nanoshells, a set of PMMA films was fabricated on Ag mirrors (without the PDMS layer) by progressively increasing the concentration of the nanoshells in PMMA solution. Figure 2(a) shows the photographs of the fabricated PMMA films for different concentrations of the nanoshells from 1% to 30%. The colors of the samples gradually changed from silver to whitish color by increasing the nanoshell concentration. The insets in Fig. 2(a) show reflection microscope images of the PMMA films, revealing large aggregations of the nanoshells. The effective diameter of the individual aggregations is several micrometers, thus being involved in the diffuse reflectance in the MIR region. At the microscopic level, concentration non-uniformity of the nanoshells was observed due to the aggregations, but they were uniformly distributed at the macroscopic level over the samples.

The reflection properties of the PMMA films were experimentally characterized by using an UV-visible-NIR spectrometer (Shimadzu, UV-3600 and MPC-3100) and a Fourier-transform infrared (FT-IR) spectrometer (JASCO, FT/IR-6300 and RF-81S). Note that an integrating sphere was used to collect the diffusively reflected light from the full solid angle in the UV-visible-NIR measurement (λ = 0.3 ~ 1.5 µm), but it was not available in the FT-IR measurement (λ = 1.5 ~ 25 µm) where only the specular reflection could be measured experimentally. Figure 2(b) shows the measured diffuse reflectance of the PMMA films in the UV-visible-NIR region. A strong absorption dips in the UV region was due to the plasma frequency of Ag, while large noises around λ = 0.9 μm were caused by the switching of an optical element in the spectrometer. The uniform diffuse reflectance was clearly observed over the visible to NIR regions, and it became higher by increasing the nanoshell concentration for stronger diffuse reflection. Figure 2(c) shows the measured specular reflectance of the PMMA films in the MIR region. By increasing the nanoshell concentration, the specular reflectance was dropped at λ < 7 μm due to the increase of the diffuse reflectance by the large aggregations of the nanoshells. This observation is also supported by comparing Fig. 2(b,c); by increasing the nanoshell concentration, the diffuse reflectance at λ = 1.5 μm in Fig. 2(b) became nearly 100%, while the specular reflectance at λ = 1.5 μm in Fig. 2(c) became nearly 0%. Many absorption dips were observed over the measurement range due to the IR absorption of PMMA and SiO_2_^18–20^. However, no major absorption dip was observed either in the solar region or the outside of the atmospheric window; therefore, the cooling performance can be comparable to the conventional devices based on the uniform diffuse reflection by the non-resonant nanoshells.

**Figure 3:**
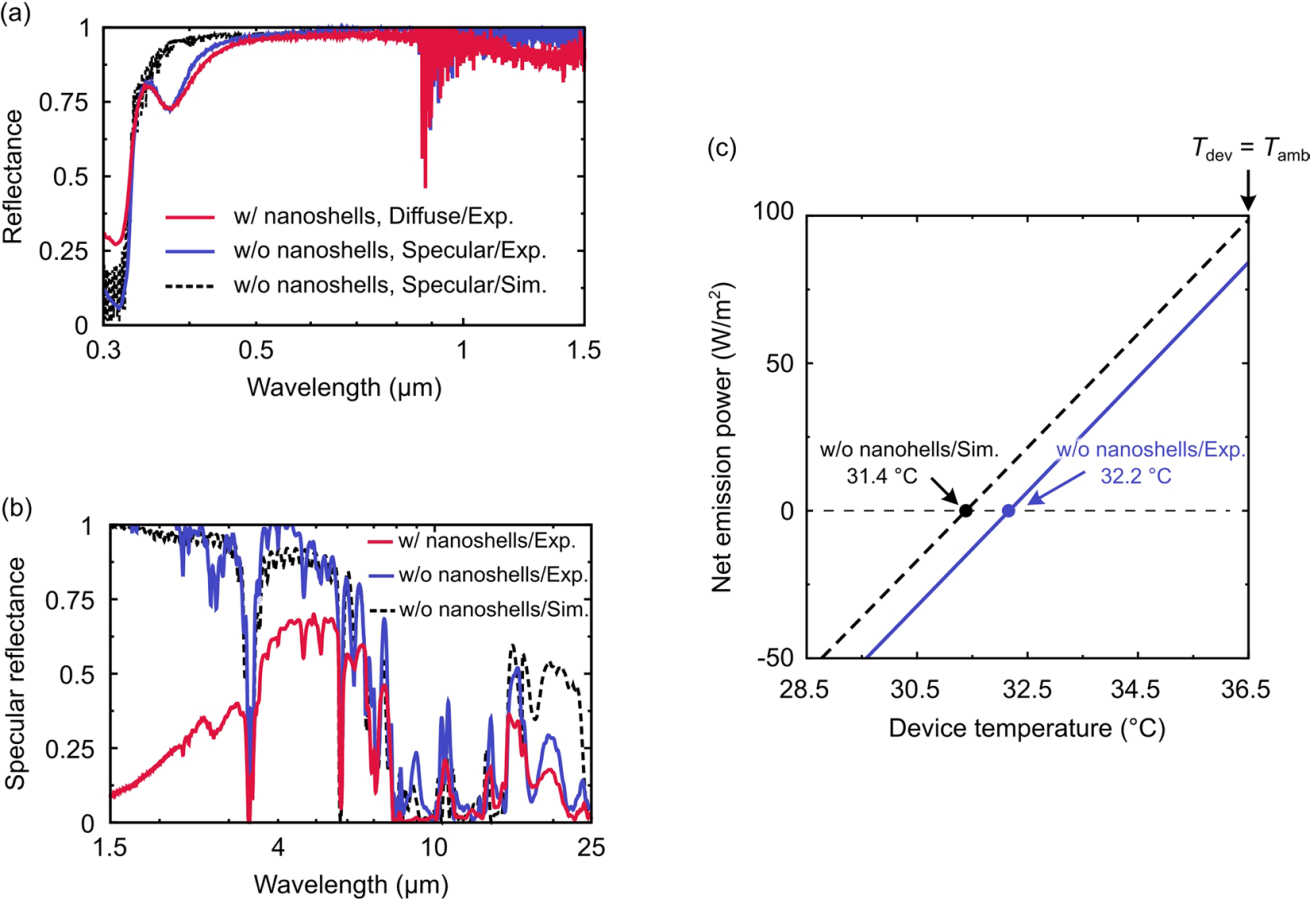
Experimentally measured (**a**) UV-visible-NIR and (**b**) MIR reflectance of the device with (red) and without (blue) the nanoshells. The experimental results without the nanoshells were well reproduced by the corresponding numerical results (dotted black). (**c**) Theoretically calculated net emission powers of the device without the nanoshells at *T*_amb_ = 36.5 °C, *E*_sun_ = 778 Wm^−2^, and PWV = 20 mm based on the measured and the simulated reflectance. The intersections of the curves with the horizontal axis of *P*_net_ = 0 represent the equilibrium temperatures, 32.2 °C and 31.4 °C, for each case.

To fully characterize the cooling device, we then evaluated the optical responses of the fabricated devices in Fig. 1(d). Figure 3(a) shows the measured diffuse (specular) reflectance of the device with (without) the nanoshells in the UV-visible-NIR region. The corresponding simulation result for the device without the nanoshells was obtained by simply solving the Fresnel equations for the multilayer structure with the empirical optical constants for PMMA, PDMS, and Ag^17,18,21–27^. In the experimental results, the device with nanoshells exhibits a uniform and high diffuse reflectance comparable to the specular reflectance of the device without nanoshells over the solar spectrum. These observations were fairly supported by the experimental results in Fig. 2(b) and the simulation result in Fig. 3(a) (dotted black), exhibiting the averaged sunlight reflectance of 0.98 over λ = 0.3 ~ 4 μm, except for absorption dips at λ = 0.38 μm in the experimental results. The discrepancy can be explained by a chemical reaction at the PDMS/Ag interface. Figure 3(b) shows the measured specular reflectance of the fabricated devices in the MIR region. A strong MIR absorption, i.e. a strong MIR radiation, was observed at λ = 8 ~ 13 μm due to an IR absorption of the PDMS layer. The device without the nanoshells has an averaged emissivity in the atmospheric window of 0.9 (Exp.) and 0.94 (Sim.), which was obtained based on Kirchhoff’s law (${\rm{\epsilon }}$ = A = 1 - R). From the Fig. 3(b), the sunlight absorption of the device with the nanoshells cannot be properly estimated due to the diffuse reflection in the MIR region. However, the cooling performance of the device with the nanoshells should be fairly the same as that without the nanoshells, because i) the decrease of the specular reflection at λ < 7 μm is due to the diffuse reflection by the nanoshells aggregations and ii) the nanoshells give no additional absorptions both in the visible and MIR (λ < 7 μm) regions.

To analyze the heat balance of the device, the equilibrium temperature was characterized by calculating the following net emission power:

1 $${P}_{{\rm{net}}}={P}_{{\rm{rad}}}({T}_{{\rm{dev}}})-{P}_{{\rm{sun}}}-{P}_{{\rm{atm}}}({T}_{{\rm{amb}}})-{P}_{{\rm{cc}}}({T}_{{\rm{dev}}},{T}_{{\rm{amb}}}),$$

where ${P}_{{\rm{rad}}}({T}_{{\rm{dev}}})$ is the radiated power from the device at the device temperature ${T}_{{\rm{dev}}}$, ${P}_{{\rm{sun}}}$ is the absorbed power from the incident sunlight, ${P}_{{\rm{atm}}}({T}_{{\rm{amb}}})$ is the absorbed power from the atmospheric thermal radiation at the ambient temperature ${T}_{{\rm{amb}}}$, and ${P}_{{\rm{cc}}}({T}_{{\rm{dev}}},{T}_{{\rm{amb}}})$ is the absorbed power from the surroundings by heat conduction and convection^7,13^.

Figure 3(c) shows the theoretically calculated net emission powers of the device without the nanoshells at *T*_amb_ = 36.5 °C by using the device’s emissivity based on the measured and simulated reflectance in Fig. 3(a,b). In the calculation, empirical values, a solar irradiance of 778 Wm^−2^, a non-radiative heat transfer coefficient of 15 Wm^−2^K^−1^, and an atmospheric transmittance at precipitable water vapor (PWV) of 20 mm were used to emulate the experimental condition^28,29^. Note that the net emission power of the device with nanoshells could not be calculated because the diffuse reflection was not available in the MIR region; however, the cooling performance can be roughly estimated from the result for the device without nanoshells. As the device temperature increased, *P*_net_ linearly increased from a negative to positive value where the device absorbs (radiates) power from (to) the surroundings at a low (high) temperature. The intersections of the curves with a horizontal axis *P*_net_ = 0 represent the equilibrium temperatures. At the *T*_dev_ = *T*_amb_, the net emission powers of each case reached 98.6 Wm^−2^ and 84.2 Wm^−2^ for the simulation and experiment, resulting in the equilibrium temperatures of 31.4 °C and 32.2 °C, respectively. Compared to the ambient temperature of 36.5 °C, the simulation result (dotted black) was 5.1 °C cooler than the ambient. The experimental result (blue) was 4.3 °C cooler than the ambient, and 0.8 °C warmer than the simulation result. This difference was due to the additional absorption dip at λ = 0.38 μm in Fig. 3(a).

The cooling performance of the device was experimentally characterized by measuring its temperature under the sunlight irradiation on a clear summer day in Okayama, Japan (35°N, 134°E, an altitude of 270 m). In the measurement, the device was placed in the visible and IR transparent wind shield, and tilted toward the sun to realize the normal incidence. The sample temperatures were measured by using thermocouples under the sample, while the ambient temperature was measured in the chamber. The detail of the measurement can be found in ref. ^30^.

**Figure 4:**
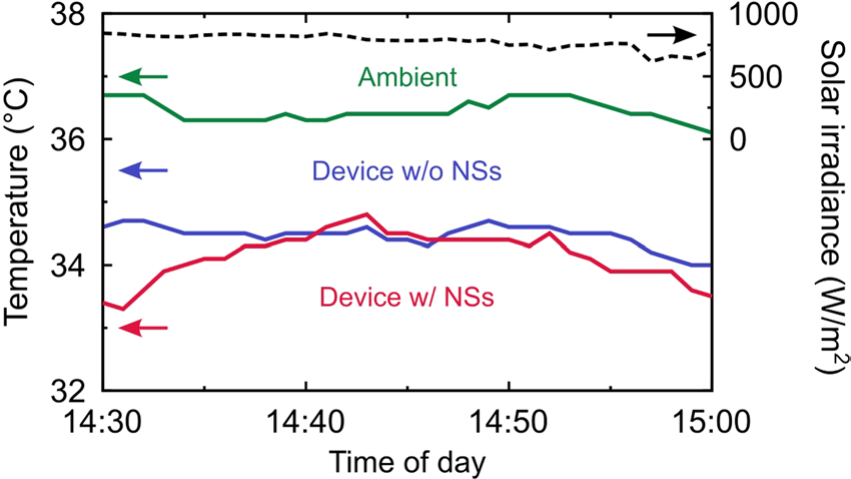
Experimentally measured temperatures of the device with (red) and without (blue) the nanoshells during the afternoon with a measurement time interval of 1 min. The measured ambient temperature (green) and solar irradiance (dotted black) is also shown for the reference.

Figure 4 shows the experimentally measured temperatures of the device with and without the nanoshells during the afternoon with a measurement time interval of 1 min. The measured solar irradiance was also shown in Fig. 4 where it showed 778 Wm^−2^ on average, gradually decreasing to the sunset. On average, the device without the nanoshells was 2 °C cooler than the ambient. Compared to the theoretical prediction in Fig. 3(c), the cooling capability was 2.3 °C deteriorated due to a high humidity in the experiment^30^. On the other hand, the device with the nanoshells was 0.3 °C cooler than the device without the nanoshells, demonstrating the comparable cooling performance compared with the conventional device as well as the whitish color by the uniform diffuse reflection of the non-resonant nanoshells.

In conclusion, the daytime radiative cooling using diffuse reflection was proposed and demonstrated. The cooling device, consisting of the PMMA layer with the randomly distributed silica nanoshells and the PDMS layer on the Ag mirror, was fabricated to exhibit uniform diffuse reflection over the solar spectrum and a selective thermal radiation in the MIR region. In the temperature measurement under the sunlight irradiation, the device showed 2.3 °C cooler than the ambient, which is comparable to or even better than the conventional device without the nanoshells. Our approach provides a simple yet powerful structure for realizing a scalable and practical daytime radiative cooling without a glaring reflective surface.
